# Modification of Lateritic Soil Using Waste Plastics for Sustainable Road Construction

**DOI:** 10.3390/polym16192689

**Published:** 2024-09-24

**Authors:** Ifeyinwa Ijeoma Obianyo, Ibitayo Akintayo Taiwo, Abubakar Dayyabu, Assia Aboubakar Mahamat, Akeem Amuda, Anthony Muoka, Abdulhameed Danjuma Mambo, Azikiwe Peter Onwualu

**Affiliations:** 1 Department of Civil Engineering, Nile University of Nigeria, Abuja 900108, Nigeria; 2 Department of Mechanical Engineering, African University of Science and Technology, Abuja 900107, Nigeria

**Keywords:** plastic waste, road construction, California bearing ratio, soil addictive, optimum moisture content, waste management, maximum dry density

## Abstract

Lateritic soil, a prevalent geological material in tropical regions, often exhibits poor engineering properties, leading to road pavement failures. Meanwhile, the alarming rise in plastic waste poses environmental concerns. This innovative study explores the potential of utilizing waste plastics as a lateritic soil addictive for sustainable road construction. Varying percentages by weight of shredded waste plastic (2%, 4%, 6%, 8%, and 10%) were incorporated into lateritic soil samples, evaluating its effects on soil geotechnical properties. The results revealed that lateritized plastic (shredded plastic waste and lateritic soil) containing 2% shredded plastic waste gave the optimum maximum dry density of 1.985 g/cm^3^, and the lateritized plastic containing 10% shredded plastic waste gave the highest optimum moisture content of 18%. However, the lower California bearing ratio obtained on the addition of plastic waste showed that the lateritized plastic is relatively weak and can only be used for roads with low traffic. The incorporation of shredded plastic waste into lateritic soil for stabilization is a promising polymer science-based method. By reducing the need for conventional materials and diverting plastic waste from landfills, this approach contributes to a more environmentally friendly infrastructure supporting the achievement of United Nation Sustainable Development Goals.

## 1. Introduction

Research estimates that more than 8.3 billion tons of plastic have been produced since the early 1950s [1]. The consumption of plastic waste poses a huge threat to the environment due to its non-biodegradability. Management of plastic waste could be very capital intensive as it requires incineration and the construction of landfills. In Nigeria, lateritic soil is commonly used for road construction. Lateritic soil in its natural stage generally has a low bearing capacity and low strength due to the high content of clay. Lateritic soil contains a large amount of clay materials; its strength and stability cannot be guaranteed under load, especially in the presence of moisture [2]. It has been found that lateritic soils are generally good construction materials and are therefore extensively used in construction [3]. Also, there are instances where lateritic soil may contain a substantial amount of clay minerals; thus, its strength and stability cannot be guaranteed under load, especially in the presence of moisture [4]. These types of laterites are also common in many tropical regions including Nigeria, where in most cases, sourcing for alternative soil may prove economically unwise but improves the available soil to meet the desired objective [5].

Since lateritic soil is prone to excessive heaving and typically falls short of the necessary density during road construction, it is known as problematic laterite when it contains the swelling clay mineral type. In theory, improving soil could involve stabilization, modification, or both. Soil modification is the addition of a modifier (cement, lime, etc.) to a soil to change the index properties. Soil stabilization, which refers to a process whereby the physical and or chemical properties of a soil are modified in order to suit the purpose a soil is meant for, has been a practice by workers that utilizes soils in construction. Over the years, cement and lime have been the two main materials used for stabilizing soil [6]. However, these materials have rapidly increased in price due to sharp increase in the cost of energy and high demand for them [7]. It has been observed also that many coal combustion by-products have properties that are beneficial in soil stabilization applications such as soil drying, the enhancement of sub-grade support capacities for pavement and floor slabs, a reduction in shrink–swell properties of soils, and improvements in aggregate road base construction and asphalt recycling [8]. Portland cement, by the nature of its chemistry, produces large quantities of CO_2_ for every ton of its final products, which contributes to the melting of the ozone layer covering the Earth’s surface [9]. Therefore, the overall environmental impact of stabilizing soil with cement can be decreased by substituting materials like plastic waste for a portion of the Portland cement used in soil stabilization.

Using lime as a stabilizer is common practice but is costly and negatively effects the environment because the chemical reactions involved are exothermic and release energy. Cement is also used as a stabilizer and it undergoes a hydration process with soil to produce a paste that bonds particles together. This process also involves energy release and is costly. There have been cases of using waste materials or residues to improve soil strength such as bone ash [10], nylon fibre [11], palm bunch ash [12], rice husk ash [13], calcium carbide residue and crumb rubber [14], calcium carbide waste, fly ash and crumb rubber [15], and waste plastic materials made from polymers like polyvinyl chloride and polyethylene terephthalate [16].

The use of shredded plastics in soil stabilization has gained attention in recent years as a sustainable and innovative solution to improve soil strength and reduce plastic waste. With the increasing global plastic pollution crisis, finding alternative uses for plastic waste has become a pressing need. Soil stabilization, a critical process in construction, agriculture, and environmental engineering, offers a potential application for shredded plastics. In Nigeria, the level of recycling of plastics is close to zero. Even in the most urbanized states such as Lagos, Abuja, and Port Harcourt, recycling is almost non-existent. Zero recycling is a poor waste management system, and the culture to properly dispose waste is lacking in the average Nigerian, resulting in Nigerian streets, gutters, roads, etc., being heavily littered. In the wake of the improper disposal and accumulation of plastics, there is a need to investigate the strength improvement of lateritic soil using plastic waste for sustainable road construction. Polyethylene terephthalate (PET) bottles are one of the most widely used plastic packaging materials globally. With the increasing concern about plastic waste management, recycling and reusing PET bottles has become a crucial area of research. Shredding PET bottles into smaller pieces has emerged as a promising approach to create value-added products. Shredded PET bottles possess unique properties that make them suitable for various applications, including construction materials, composites, and packaging. Understanding these properties is crucial for optimizing their use and developing sustainable solutions for plastic waste management. According to a study by some researchers, waste PET plastic can be chemically recycled into useful additives that improve rubberized bitumen’s overall performance [17]. The study’s authors also claimed that the recycling technique they developed helps to reduce the problems associated with disposing of waste PET plastic and scrap tires in landfills by converting them into valuable new materials that can be used to construct long-lasting pavements. Mechanical recycling is the most popular technology for PET recycling, with benefits including low energy consumption, low cost, and high-quality output, according to a recent review that looked at several PET recycling methods, including chemical, enzymatic, mechanical, and pyrolysis [18]. However, there are limited studies on the utilization of PET for the stabilization of lateritic soil for sustainable road construction. The use of shredding PET bottles plastic waste as an additive for soil stabilization was examined in this study. The mechanical characteristics of lateritic soil treated with shredding PET bottles plastic waste were studied, and the physical characteristics of the raw materials employed in the study were evaluated to obtain the best percentage of plastic waste admixture for lateritic soil strength improvement. The utilization of plastic waste in the treatment of laterite is eco-friendly and also cleans up the environment while improving the strength of lateritic soil. Plastic waste could be more viable as a stabilizer because it does not react with soil and thus requires no energy release to function as a stabilizer [19]. This research pioneers a new frontier in waste plastic utilization, paving the way for innovative, eco-friendly road construction practices that support the circular economy and mitigate environmental pollution. By incorporating shredded plastics into soil, it is possible to enhance soil properties, reduce erosion, and increase durability [20]. This approach not only addresses soil stability issues but also provides a viable solution for managing plastic waste, promoting a circular economy, and mitigating environmental pollution.

## 2. Utilization of Shredded Plastics in Soil Stabilization

### 2.1. Properties of Shredded PET (Polyethylene Terephthalate) Bottles

Shredded plastic wastes are generated from post-consumer plastic materials, such as polyethylene, polypropylene, and polyvinyl chloride (PVC). These plastics are shredded into small pieces, typically ranging from 2 to 5 mm in size [21]. The physical properties of shredded plastic wastes, including density, moisture content, and particle size distribution, vary depending on the type of plastic and shredding process [22]. PET, or polyethylene terephthalate, is a kind of polyester polymer resin that is often coded on or next to the bottom of bottles and other containers. PET has a number of crucial qualities, including strength, transparency and gas barrier qualities. Shredded plastic wastes exhibit unique properties that make them suitable for soil stabilization. These properties can be classified into physical, mechanical, thermal and chemical properties.

The unique physical properties of shredded PET bottles include particle size and distribution ranging from 1 to 10 mm in size, with a median size of 3–4 mm [23,24]; density between 1.38 and 1.40 g/cm^3^, similar to that of virgin PET [25,26]; and low moisture content, ranging from 0.1 to 0.5% [23,27]. The mechanical properties of shredded PET bottles are critical for their potential applications. The flexural strength ranges from 30 to 60 MPa [28] and they have moderate impact resistance, with an Izod impact strength of 20–50 J/m [29]. Understanding the thermal properties of shredded PET bottles is essential for its processing and application. The thermal properties of shredded PET bottles include a melting point of around 250–260 °C, glass transition temperature of approximately 70–80 °C, and a thermal conductivity of 0.2–0.5 W/mK [30]. The chemical properties of shredded PET bottles are vital for their compatibility and durability. These properties include excellent resistance to water, acids, and bases and degradation when exposed to UV light, heat, or chemicals [31].

### 2.2. PET (Polyethylene Terephthalate) Bottle Polymerization Method

Bottles made of polyethylene terephthalate (PET) are frequently used to package food, drinks, and personal hygiene items. Making PET bottles entails a complex polymerization process, which transforms raw materials into a high-quality plastic material. This article delves into the PET bottle polymerization method, exploring the raw materials, reaction mechanisms, process conditions, and product properties. The PET bottle polymerization process begins with two primary raw materials: Ethylene glycol (EG), which is a colourless, odourless liquid used as the diol component, and Terephthalic acid (PTA), which is a white, crystalline powder used as the dicarboxylic acid component [32]. The PET polymerization process involves a condensation reaction between EG and PTA, resulting in the formation of PET polymer chains. The reaction mechanisms can be divided into three stages: esterification, where EG and PTA react to form bis(2-hydroxyethyl) terephthalate (BHET); transesterification, where BHET reacts with additional PTA to form PET polymer chains; and polycondensation, where PET polymer chains undergo further condensation to increase molecular weight [32]. The PET bottle polymerization process is conducted under specific conditions such as a temperature range of 250–300 °C, pressure range of 2–5 bar, commonly used catalysts of antimony trioxide or germanium dioxide, and residence time range of 2–5 h [33]. The resulting PET polymer exhibits unique properties such as a molecular weight of 10,000–50,000 g/mol, intrinsic viscosity of 0.8–1.2 dL/g, melting point of 250–260 °C, and glass transition temperature of 70–80 °C [30].

The PET bottle polymerization method is a complex process involving raw material selection, reaction mechanisms, process conditions, and product properties. Understanding this process is crucial for optimizing PET production, improving product quality, and reducing environmental impact.

### 2.3. Mechanisms of PET Plastic Waste-Based Soil Stabilization

Soil stabilization is a critical process in construction, agriculture, and environmental engineering to improve soil strength, reduce erosion, and increase durability. Recently, plastic waste-based soil stabilization has gained attention as a sustainable solution [32]. Shredded plastic waste, typically polyethylene or polypropylene, is mixed with soil to create a composite material. The plastic particles interact with the soil matrix, forming a network of polymer chains that enhance soil strength and stability. The PET–soil interaction can be attributed to the following mechanisms:

- Mechanical Interlocking: PET particles fill soil voids, increasing soil density and reducing settlement [22]
- Polymer–Soil Adhesion: Polymer chains in shredded plastics bond with soil particles, increasing soil cohesion [34].
- Hydrophobic Behavior: The interface between shredded plastics and soil particles influences soil properties, such as water retention and permeability [35]
- Chemical Bonding: PET particles react with soil minerals, forming chemical bonds that improve soil stability [36].

The size and content of PET particles significantly impact the stabilization mechanisms. Smaller PET particles (0.1–1 mm) exhibit higher surface area, increasing polymer–soil adhesion and mechanical interlocking [37]. Optimal PET content (5–15% by weight) balances mechanical interlocking and polymer–soil adhesion, maximizing soil stability [32].

Soil properties, such as texture, structure, and mineralogy, affect the PET–soil interaction. PET particles interact more effectively with sandy soils than clayey soils due to increased surface area [38]. PET particles reinforce soil aggregates, improving soil structure and stability. PET particles react with soil minerals, such as calcium and silica, forming chemical bonds that enhance soil stability [39].

The mechanisms of PET bottle plastic waste-based soil stabilization involve complex interactions between PET particles and soil matrices. Understanding these mechanisms is crucial for optimizing PET content, particle size, and soil properties to achieve effective soil stabilization. The polymer science behind using shredded plastic waste for soil stabilization presents a groundbreaking solution for sustainable construction and environmental engineering. By understanding the mechanisms and benefits of this method, we can unlock its full potential and develop innovative applications to address global challenges

### 2.4. Benefits of Plastic Waste-Based Soil Stabilization

Plastic waste-based soil stabilization is a method of soil stabilization that offers several advantages over traditional soil stabilization techniques. These advantages include the following:

- Sustainability: Plastic waste-based soil stabilization utilizes non-biodegradable plastic waste, reducing landfill disposal and environmental pollution.
- Cost-effective: Shredded plastic waste is often readily available and inexpensive.
- Improved soil strength: Enhanced mechanical properties, including increased compressive strength and reduced settlement.

The use of shredded plastic waste for soil stabilization has far-reaching applications in the field of construction, where they are used for road construction, foundation engineering, and building materials, and field of environmental engineering, where they are used for landfill capping, contaminated soil remediation, and coastal protection. The incorporation of shredded plastic wastes into lateritic soils can significantly improve the soil by minimizing water-induced soil instability and erosion, enhancing the compressive strength and shear strength of lateritic soils, and reducing soil erosion and increasing resistance to weathering [40].

### 2.5. Soil Classification and Stabilization

In the context of civil engineering, soil refers to naturally occurring, loose, uncemented, or weakly unconsolidated mineral particles that can be either organic or inorganic. Soil is found on top of bedrock, which is created when rocks gradually break down. Particles including gravel, rock, sand, silt, clay, loam, and humus combine to produce soil. The classification of soil allows geotechnical engineers to accurately predict their properties and behaviour by grouping them into similar response categories based on index properties. On building sites, one can come across a variety of soil kinds. Soil surveys are frequently necessary to support design and construction decisions because of the significance of the features of soil, including the size and kind of particles, its density, and its structural qualities. Using a standard classification method, a soil survey will classify the soil, map out the boundaries of different soil types, and forecast the soil’s expected behaviours [41].

The process of preserving or changing one or more soil attributes in order to enhance a soil’s performance and engineering qualities is known as soil stabilization. In general, stabilization refers to the various ways that soil characteristics can be changed to increase the soil’s capacity for engineering.

#### 2.5.1. Common Stabilization Processes

The term “soil stabilization process” refers to a group of methods used to modify the mechanical and/or physical characteristics of soil for a given use. It might also entail compacting the soil to strengthen its resilience to loading, adding organic binders like cement to strengthen and last, or, in the worst-case situation, repurposing unsuitable material [42].

Various methods for soil stabilization exist, such as mechanical, electrical, and additive stabilization. Mechanical methods are the most ancient forms of soil stabilization. Mechanical soil stabilization involves physically changing the property of the soil by compaction with rollers and plate compactors in order to affect its gradation, solidity, and other characteristics. Another method is by additives. Additives are substances added to something in small quantities to improve it and are usually chemical in nature.

Chemical soil stabilization is used as an alternate means of soil stabilization. This method depends on incorporating extra materials into the soil, which will interact with it and alter its characteristics. Sodium silicate, calcium chloride, and sodium chloride are the three most prevalent chemical compounds. Common stabilizers used in practice are also chemical in nature. These are cement, fly ash, and lime. Lime also helps to remove water from the soil, which allows it to compact more [43].

#### 2.5.2. Plastic Waste Stabilizers Compared to Other Stabilizers

In terms of the economy, plastic will act as a cheaper option for soil improvement because other methods either require equipment or purchasing the additives, which costs more than plastic because it is available as a waste product. In terms of time consumption, the effects of plastic waste and mechanical and electrical soil improvement will be immediate, while the methods involving additives will require time for reactions to occur within the soil. In terms of environmental effects, lime, cement, fly ash, and chemical compounds will undergo reactions with the soil that produce heat and release carbon dioxide into the environment while plastic waste soil improvement brings no harm

### 2.6. Soil Geotechnical Properties for Construction

#### 2.6.1. Compressibility Properties

A soil might be classified as low strength if it is weak/compressible, collapsible, or expansive. Soil compressibility occurs at a lesser degree when it is more coarse and less aggregated, and thus, generally, gravelly soils are less compressible than fine soils [44]. Collapsible soils are soils that seem to be strong/stable in their dry state, but quickly consolidate when wet, generating large settlements. Expansive clay soils are types of soils whose volumes experience significant change with a change in their moisture content. A recent study investigated the outcome of stabilizing expansive clayey soils with an additive form of stabilization with the using strips from plastic bottle [45].

#### 2.6.2. Soil Strength Properties

Some properties that are looked at when determining soil strength are the soil structure, water content, cohesion, and angle of internal friction. Soil structure refers to the particle size, shape, arrangement and presence of voids; the continuity of pores and their capacity to retain and transmit fluids and organic and inorganic substances, and their ability to support intense root growth and development, is also included. The nature of the soil structure affects soil strength parameters. The description of soil structure in the field highly depends on soil moisture content, especially in swell-shrinking soils [46].

## 3. Materials and Methods

### 3.1. Materials

**Figure 1:**
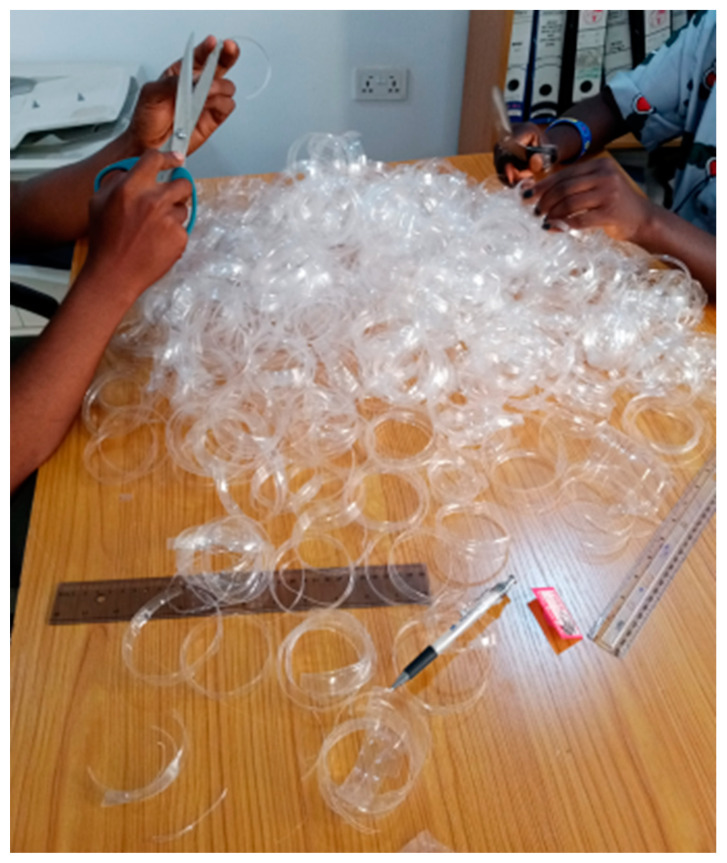
Shredded plastics from PET bottles.

The materials used for this study were the lateritic soil sample and shredded plastics. The soil used in this research was obtained from Federal Capital Abuja (Guzape District, Nigeria). The stabilizer used in this research was shredded plastics obtained from PET bottles, as shown in Figure 1. The dimensions of the shredded PET strips used for this study are 10 × 50 mm. Clean water was used for the mixing of the lateritic soil and the shredded plastic waste.

### 3.2. Preparation of Plastic-Soil Mixtures (Lateritized Plastic)

**Figure 2:**
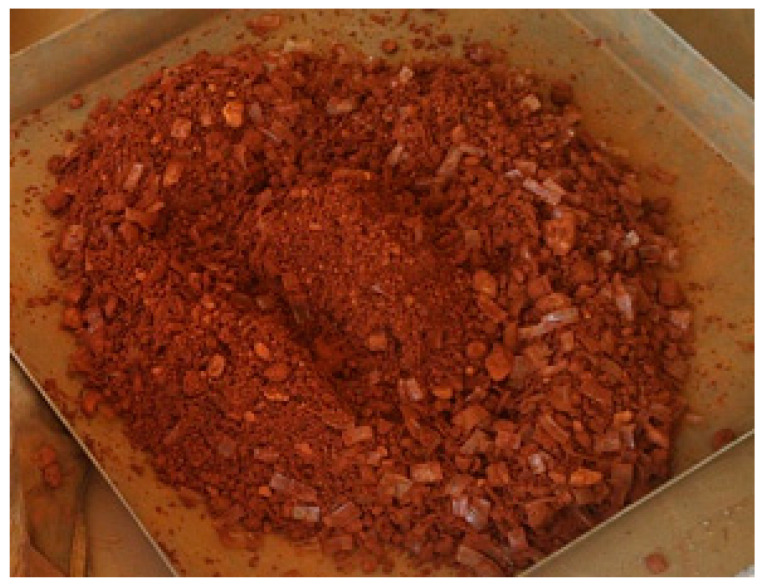
Lateritized plastic (mixture of lateritic soil and shredded plastic waste).

The materials needed for preparing lateritized plastic include lateritic soil, shredded PET (from plastic bottles), water, a mixing container, a trowel for mixing, and a sieve. The following procedures should be followed in order to prepare a mixture of lateritic soil and shredded PET (polyethylene terephthalate): Choose a suitable supply of lateritic soil and dig it up. Sieve the soil to remove any rocks, debris, or vegetation. The PET material should be chopped or shredded into tiny pieces, ideally 1 to 5 mm in size. Decide on the proportion of lateritic soil to shredded PET (2%, 4%, 6%, 8%, and 10% of shredded PET was used for this investigation). In the mixing container, mix the shredded PET with lateritic soil as shown in Figure 2. Prior to mixing, make sure the PET shreds are dry and clean. As you mix, progressively add additional materials after starting with less. Water should be added gradually to the mixture while stirring. The recommended range for moisture content is 10–15%. Mixing should be performed until the mixture is homogenous and the materials are well blended.

### 3.3. Tests

To characterize the unreinforced soil sample, indicator tests are performed on it. The pycnometer test is used to measure soil density, while the Atterberg limit test is used to calculate the plastic limit, liquid limit, and plasticity index. To find out if adding plastic trash improved or worsened the soil engineering properties, more tests were conducted on both stabilized and unstabilized samples. The Proctor test was used to evaluate soil compaction in order to determine the point at which soils may be compacted with the greatest efficiency using construction equipment. This was performed based on the maximum dry density and optimal moisture content of the soil.

#### 3.3.1. Atterberg Limits

The Atterberg limits, also referred to as the liquid limit (LL) and plastic limit (PL), are the most often used tests prescribed by geotechnical engineers in practice worldwide. They have physical significance for fine-grained mineral soils. In addition to being connected with several basic soil metrics utilized in design and construction projects, they are used to classify soils. The water content, or LL, indicates the point at which the solid particles separate from one another and the soil starts to flow and behave like a liquid. The ductile–brittle transition, or the limit of soil workability, is defined by the standard PL (thread rolling). The Atterberg limit test was conducted in compliance with [47].

#### 3.3.2. Sieve Analysis

The proportions of the different particle sizes that a soil contains are expressed by its particle size distribution. The relative weights of certain size classes or the relative numbers of particles within particular size classes are used to describe the proportions. The sieve analysis test was carried out in compliance with [48].

#### 3.3.3. Specific Gravity

The specific gravity of fine-grained soil solids (GS) is a crucial feature of soil grains that is utilized in many analyses, including sedimentation, compaction, and consolidation, to extract other properties including void ratio, degree of saturation, and soil densities. As specified by [49], the Specific Gravity Test was conducted.

#### 3.3.4. Proctor Compaction Test

The purpose of compacting earth fills is to produce a soil mass that will satisfy three basic criteria: settlement reduction, permeability reduction, and an increase in shear strength. Through the proctor compaction test, two important compaction characteristics, maximum dry density (MDD) and the optimum moisture content (OMC), are obtained, with a relationship established between them. The proctor compaction test was performed with reference to [47].

#### 3.3.5. California Bearing Ratio Test

The California bearing ratio (CBR) is the ratio of the resistance to sinking in a standard crushed rock sample at the same rate for the same penetration depth and the resistance against sinking of a penetration plunger into soil compacted at a specific energy in a predetermined moisture content at a rate of 1.27 mm/min velocity. In compliance with [47], the test was carried out.

### 3.4. Preliminary Analysis Results

#### 3.4.1. Chemical Composition of Lateritic Soil

The chemical composition of the lateritic soil sample used for this research obtained using X-ray Fluorescence (XRF) is presented in Table 1. The result shows that the lateritic soil sample used for this study is not pozzolanic materials as the summation of oxides of silicon, aluminium, and iron is less than 70% [12]. Oxides present in the sample are predominantly SiO_2_, Al_2_O_3_, Fe_2_O_3_, K_2_O, and MgO.

#### 3.4.2. Sieve Analysis of Lateritic Soil (Wet/Dry Sieving)

**Figure 3:**
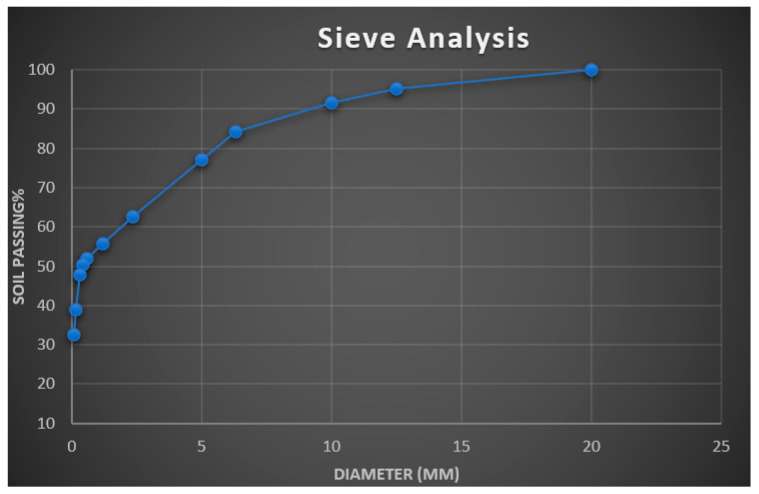
Particle size distribution of lateritic soil.

The sieve analysis of lateritic soil used to assess if a sample complies with design, production control requirements, and verification criteria by grading (the distribution of aggregate particles, by size) is shown in Figure 3. It can be deduced that the lateritic soil material is gap-graded, which represents a soil with a combination of two or more uniformly graded fractions. The particle distribution curve can be used to determine the proportion of gravel, sand, silt, and clay size particles in soil. Since 32.6% of the soil passes through the No 200 sieve, the soil sample is a fine-grained soil.

#### 3.4.3. Specific Gravity Test/Relative Density

Specific Gravity Test is also known as Relative Density. The Relative Density of lateritic soil is shown in Table 2. This can be expressed as the ratio of a substance’s density to that of a certain reference material, such as water. The specific gravity of the tested lateritic soil was found to be 2.52.

#### 3.4.4. Atterberg Limit

The result of Atterberg limits test of lateritic soil is presented in Table 3. The soil sample used can be classified as silts and clay using the Unified Soil Classification System since LL (liquid limit) < 50% and CL since PI > 7. The lateritic soil used for this study is classified as soil with low plasticity since liquid limit (LL) is 34%, which is less 50%.

**Figure 4:**
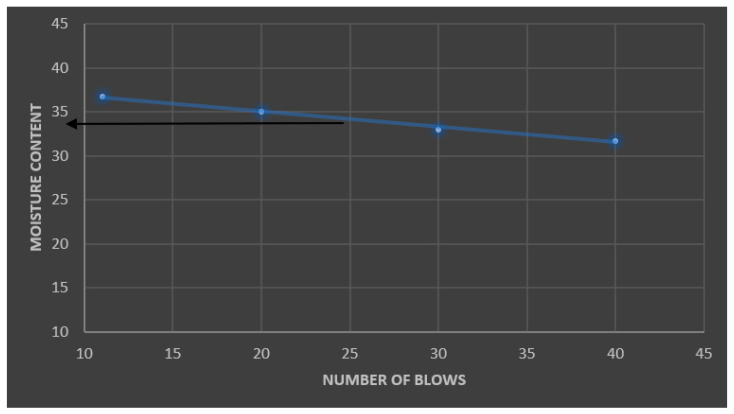
Flow curve for liquid limit determination for lateritic soil (the arrow indicates the liquid limit of the lateritic soil used).

The graph in Figure 4 reflects the relationship between the moisture content and the number of blows. The liquid limit of the soil material used in the experiment was obtained at 25 blows. The liquid limit of the soil material used is 34%.

## 4. Results and Discussions

### 4.1. Proctor Compaction Test Results

**Figure 5:**
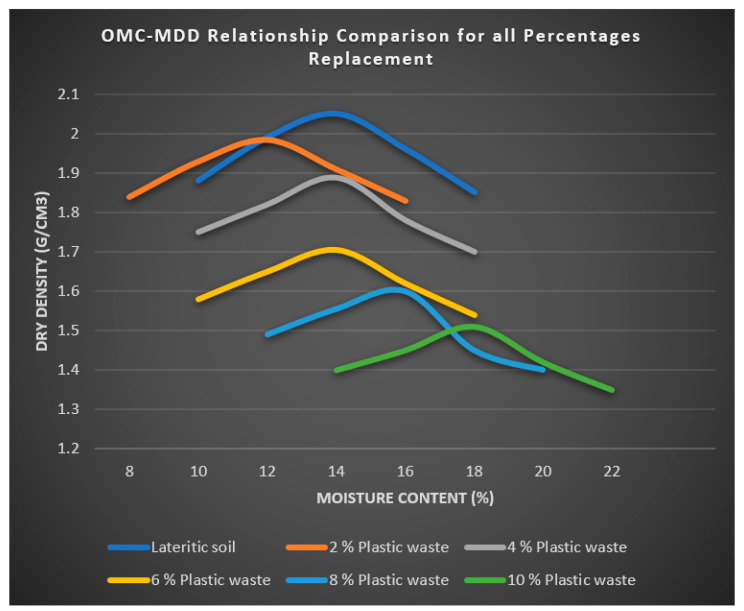
OMC-MDD relationship comparison for all percentages replacement.

The result presented in Figure 5 revealed that the optimum moisture content (OMC) of 14% and maximum dry density (MDD) of 2.05 g/cm^3^ were obtained for the lateritic soil used with 0% of plastic waste fibre. There is a reduction in the value of OMC to 12% and that of MDD to 1.985 g/cm^3^ of the lateritized plastic containing 2% plastic waste fibre. This reduction is due to resulting permeability and capillary action of the composite material with the addition of plastic waste fibre. As the percentage of plastic waste fibre increases to 4%, the value of OMC increases to 14% due to more space being occupied by the plastic waste fibre, on which moisture stays on the surfaces of the plastic fibre. However, the MDD further decreases to 1.888 g/cm^3^ due to the increase in the proportion of the shredded plastic waste. The value of OMC remains unchanged as the plastic waste fibre content increases from 4% to 6% in the lateritized plastic. However, the MDD was found to further decrease to 1.705 g/cm^3^. As the percentage of plastic waste fibre increased to 8% in the composite (lateritized plastic), the OMC increased to 16% while the MDD decreased to 1.600 g/cm^3^ as the lighter plastic waste proportion increased. The OMC, as presented in Figure 5, was achieved at 18%, as plastic waste fibre increased to 10% and a reduced maximum dry density of 1.510 g/cm^3^ was obtained due to the light weight of the shredded plastic waste. The reduced soil density is used in engineering to build lightweight embankments. The more the shredded plastic waste is added to the lateritized plastic, the lighter the resulting composite. This kind of composite is appropriate for applications requiring a lower maximum dry density [47].

The range of the OMC for lateritic soil to mixtures of laterite with plastic waste fibre (2%, 4%, 6%, 8%, and 10%) is 14% to 18%, as shown in Figure 5. The plastic waste fibres that act as soil filler were the cause of the increases in the OMC. The MDD, which ranges from 2.05 gm/cm^3^ to 1.5 gm/cm^3^, falls as the amount of plastic waste fibre in the lateritic soil increases, as presented in Figure 5. The best results for MDD and OMC were achieved when lateritic soil was substituted for plastic waste fibre at 2% and 10%, respectively. The results, as shown in Figure 5, also showed that the lateritic soil utilized with 0% of plastic waste fibre produced an optimal moisture content of 14% and a maximum dry density of 2.05 g/cm^3^.

### 4.2. California Bearing Ratio (CBR)

**Figure 6:**
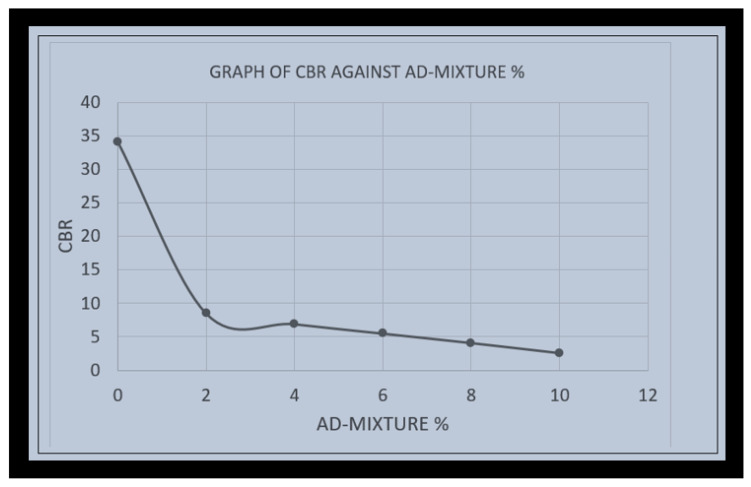
CBR against the percentage of admixture.

The CBR of various samples of lateritic soil and composite material of shredded plastic waste bottle of various percentage of 0%, 2%, 4%, 6%, 8%, and 10% by weight were tested, and the results of the CBR of lateritic soil with zero percent and different percentage of plastic waste fibre are presented in Figure 6. The CBR of lateritic soil with zero percent plastic waste fibre was 34.1, which was achieved by consolidation, serving as a reference for various percentages of the admixture of the plastic waste fibre used in the experiment. The CBR reduced to 8.5, 6.9, 5.5, and 4.1 with the addition of 2%, 4%, 6%, and 8% of plastic waste fibre admixture, respectively, due to the presence of the admixture that reduce the penetration of the plunger. The results presented in Figure 6 show that the addition of the 10% plastic waste fibre admixture resulted in a reduced penetration of the plunger, leading to a further reduction in the CBR value to 2.6.

## 5. Conclusions

This research investigated the use of shredded plastic waste for the effective strength improvement of lateritic soil for sustainable road construction. In order to achieve this objective, varying percentages by the weight of shredded plastic from 2%, 4%, 6%, 8%m and 10% were used to replace the lateritic soil used for the experiment, and the resulting lateritized plastic (shredded plastic waste and lateritic soil) were compared with lateritic soil to determine the optimum percentage for strength improvement. The following conclusions were made from this study:

▪ The OMC from lateritic soil to (2%, 4%, 6%, 8%, and 10%) the mixture of laterite with plastic waste fibre ranges from 14 to 18%. The increases in the OMC were due to the effect of plastic waste fibres, which serve as a filler in the soil.
▪ The MDD decreases as the plastic waste fibre content increases in the lateritic soil, with the value ranging from 2.05 gm/cm^3^ to 1.5 gm/cm^3^. The optimum value for both MDD and OMC were obtained at a 2% and 10% plastic waste fibre replacement with lateritic soil, respectively.
▪ As the percentage of shredded plastic waste increases from 2% up to 10% in the composite material (lateritized plastic), the value of CBR decreases, with lateritized plastic containing 2% plastic waste fibre having the optimum value of CBR.
▪ From the compaction test, the addition of shredded plastic waste resulted in a composite material (lateritized plastic) with a lower weight and density. This composite can be used for light weight structures or road pavement with medium traffic.
▪ Furthermore, the use of waste plastic serves as an effective means of recycling and repurposing plastic waste materials, contributing to environmental sustainability. Incorporating shredded plastic wastes into lateritic soil for stabilization is a promising technique rooted in polymer science. Understanding the interactions between shredded plastics and soil particles, and the properties of both materials, is crucial for optimizing this method. Further research is necessary to explore the long-term effects and environmental implications of this technique.

## Figures and Tables

**Table 1:** Chemical oxides in lateritic soil.

Oxides	SiO_2_	Al_2_O_3_	Fe_2_O_3_	K_2_O	MgO	TiO_2_	BaO	Sb_2_O_3_	MnO	CdO	CaO
**%**	29.16	19.25	7.29	3.43	2.53	1.07	1.00	1.00	0.67	0.14	0.11

**Table 2:** Determination of specific gravity of lateritic soil by pycnometer method.

		TEST	1	2
a	Weight of bottle	w1	650.3	650.3
b	Weight of bottle + water	w2	1961.2	1961.2
c	Weight of bottle + sample	w3	1650.1	1650.1
d	Weight of bottle + water + sample	w4	2564.1	2564.1
e	Weight of water to full bottle: w2–w1	a	1310.9	1310.9
f	Weight of sample: w3–w1	b	999.8	999.8
g	Weight of water added to sample: w4–w3	c	914	913.9
h	Weight of water displaced by sample: a–c	d	396.9	396.9
i	Specific gravity: b/d		2.52	2.52
	Average		2.52	

**Table 3:** Atterberg limits test results for lateritic soil.

LL	PL	PI
34%	23%	11%

## Data Availability

The data that support the findings of this study are available from the corresponding author, I.I.O., upon reasonable request.
